# Breastfeeding Communication Strategies, Challenges and Opportunities in the Twitter-Verse: Perspectives of Influencers and Social Network Analysis

**DOI:** 10.3390/ijerph18126181

**Published:** 2021-06-08

**Authors:** Sara Moukarzel, Anita Caduff, Martin Rehm, Miguel del Fresno, Rafael Pérez-Escamilla, Alan J. Daly

**Affiliations:** 1 Larsson-Rosenquist Foundation Mother-Milk-Infant Center of Research Excellence, University of California San Diego, La Jolla, CA 92161, USA; 2 Department of Education Studies, University of California San Diego, La Jolla, CA 92161, USA; acaduff@ucsd.edu (A.C.); ajdaly@ucsd.edu (A.J.D.); 3 Institute of Educational Consulting, University of Education Weingarten, 88250 Weingarten, Germany; martin.rehm@ymail.com; 4 Department of Social Work, National Distance Education University, 28015 Madrid, Spain; delfresno@gmail.com; 5 Department of Social and Behavioral Sciences, Yale School of Public Health, New Haven, CT 06510, USA; rafael.perez-escamilla@yale.edu

**Keywords:** breastfeeding, public health, social media, social network analysis, science communication, communication strategies, influencers, Twitter, challenges and opportunities

## Abstract

Using social media is one important strategy to communicate research and public health guidelines to the scientific community and general public. Empirical evidence about which communication strategies are effective around breastfeeding messaging is scarce. To fill this gap, we aimed to identify influencers in the largest available Twitter database using social network analysis (*n* = 10,694 users), inductively analyze tweets, and explore communication strategies, motivations, and challenges via semi-structured interviews. Influencers had diverse backgrounds within and beyond the scientific health community (SHC; 42.7%): 54.7% were from the general public and 3% were companies. SHC contributed to most of the tweets (*n* = 798 tweets), disseminating guidelines and research findings more frequently than others (*p* < 0.001). Influencers from the general community mostly tweeted opinions regarding the current state of breastfeeding research and advocacy. Interviewees provided practical strategies (e.g., preferred visuals, tone, and writing style) to achieve personal and societal goals including career opportunities, community support, and improved breastfeeding practices. Complex challenges that need to be addressed were identified. Ideological differences regarding infant feeding may be hampering constructive communication, including differences in influencers’ interpretation of the WHO International Code of Marketing of Breast-milk Substitutes and in perspectives regarding which social media interactions encompass conflict of interest.

## 1. Introduction

The overall mission of public health is to protect and promote the health of all people in all communities. This mission became highly visible during the COVID-19 pandemic. One of the well-recognized frameworks to achieve this mission is the 10 Essential Public Health Services (EPHS) [1]. Centered around equity, EPHS incorporate key health services highly relevant to current and future public health practice, in the United States and around the globe (Supplementary Materials Figure S1). One of these services is “to communicate effectively to inform and educate people about health, factors that influence it, and how to improve it”. To achieve effective communication, the 10 EPHS taskforce recommends several approaches, such as communicating with accuracy and necessary speed and using appropriate channels to reach intended audiences (i.e., using social media and peer-to-peer networks) [1]. However, we still lack empirical research around how these approaches are being executed. 

The robust data generated by us and others suggest social media spaces are becoming important key players in shaping conversations around public health among and across members of the general public, the scientific community, health organizations, and commercial companies [2,3,4,5]. Many of these conversations are driven or amplified by identifiable “virtual influencers” who connect users together and who become opinion leaders and sources of authority, even if the content they share is not necessarily accurate [6,7,8,9]. These influencers may be identified based on their real-time social interactions on social media (e.g., replying, mentioning, or being mentioned on Twitter), instead of assuming influence based on number of followers [3,10,11]. These influencers may provide important insights into what strategies work best when communicating about public health on social media—an understudied area of research [12]. This paper uses “discussions about breastfeeding on Twitter” as a case study to showcase lessons learned when examining the dynamics of online social networks. Breastfeeding, as an exemplar case, is relevant as the death of 800,000 infants and 20,000 mothers may be prevented annually with universal, exclusive, and sustained breastfeeding [13]. Twitter has been our focus due to its increased popularity among diverse users globally and the relative ease of collecting publicly available user profile information and activity in real time [14].

The term “social network” is often conflated with social media, but the terms are not synonymous. Rather, a *social network* refers to a group of individuals who interact together, communicating offline or on social media about a common larger theme [15]. For example, a group of Twitter users who communicate together about a particular topic (e.g., breastfeeding benefits) via tweets, retweets, or comments may be referred to as a “social network on Twitter around breastfeeding benefits”. It is well established that individuals influence and are influenced by the social networks in which they are embedded [16,17]. Social networks are important as they allow the flow and dissemination of diverse information, opinions, attitudes, norms, and forms of social support. Therefore, social networks can influence our understanding, intentions, decisions, and ultimately health behaviors [17,18].

Social network analysis (SNA) focuses on measuring and understanding the social interactions between users, rather than focusing on individual-level attributes [15]. To date, we have shown that SNA of Twitter data can help inform public health communication strategies in more nuanced ways than simply reviewing Twitter Analytics metrics (e.g., number of times a tweet was retweeted, number of followers, number of “likes”). In 2020, we cross-sectionally identified influencers who dominated breastfeeding conversations [3,19], when COVID-19 breastfeeding guidelines were released [10], and during World Breastfeeding Week [2]. What remains missing in the literature is a better understanding of what communication strategies contribute to users being influencers in the public health sphere. In digital advertising and marketing for commercial purposes, there is plenty of information about what consumers want and which strategies attract and retain clients (i.e, type of content, visuals, tone, posting frequency, level of engagement with users etc.) [20]. However, this has not been the case in public health. Hence, the goal of this study was to (a) identify influencers in the most extensive database available to date related to breastfeeding conversations on Twitter, (b) to analyze their tweets, and (c) to better understand their communication strategies, motivations, and challenges to reach their communication goals. We expect that this research will help identify strategies to enhance audience reach and uptake of messages shared by public health professionals on social media.

## 2. Materials and Methods

This mixed-methods study included a quantitative SNA to identify Twitter influencers, and a qualitative inductive approach to describe the influencers’ backgrounds and tweet content, as well as communication strategies. First, we accessed Twitter’s application programming interface as permitted by the platform’s terms and conditions and collected all breastfeeding-related tweets and associated user profile information over a duration of six months from 1 January to 1 July 2020 [2,3,19]. We targeted tweets which included at least one of the following commonly used hashtags: #breastfeed, #breastfeeding, #normalizebreastfeeding, #Breastfeeding, #breastmilk, #breastfeedingmoms, and #breastfeedingsupport [19]. The resulting database consisted of 14,214 tweets including 10,694 users who had accessible profile information, such as profile description, number of followers, profile picture, and location. Tweet information included time stamp, content, username of sender, and type (tweet or retweet).

To identify influencers who had disproportionate influence over conversations, SNA was conducted and overall degree was calculated for each user, as described in depth in our previous work [6]. Briefly, a sociogram, which illustrates breastfeeding-related interactions, was produced using the open-source network analysis and visualization software package Gephi (version 0.9.2 for Mac, 2017). Then, *overall degree* was calculated as the sum of *outdegree* (number of times a user mentions or retweets another user) and *indegree* (number of times a user is mentioned or retweeted) also using Gephi [21]. Users in the top 10% by overall degree who had accessible profile information and whose accounts were active at the time of data analysis were identified as influencers (256 users did not have accessible data) [6,21]. These influencers contributed to a total of 1373 tweets/retweets.

Second, to describe the background of influencers, we used inductive coding as previously carried out [2,3,22]. Two researchers first independently and then together examined influencers’ profiles and tweeting history to develop a codebook (Table S1). To ensure accuracy in coding, we verified influencers’ claimed professional credentials and affiliations using academic, clinical, governmental, or non-governmental agency websites. Tweets were similarly categorized using inductive coding and were cross-checked by a third researcher (Table S2 for codebook). To ensure accuracy of tweet coding, we also reviewed all photos and external links attached to a tweet. 

Third, to elucidate tweeting strategies that contributed to being influential in the breastfeeding conversation on Twitter, we undertook a deeper examination of the influencer database and identified those who were in the top 10% by overall degree *for four or more weeks over the six-month data collection period* (*n* = 35). The four or more weeks was not a pre-set criterion but rather chosen based on our observations that the predominant majority of influencers (>97%) did not maintain influential status for more than a month. We then invited these *top* influencers via email or Twitter direct messaging to participate in a voice-recorded semi-structured interview using Zoom (Table S3 for sample questions). Twenty interviews were conducted lasting 18–49 min, after which interviews were transcribed verbatim. Then, two researchers independently read the transcripts to familiarize themselves with the content and, together, they used open-coding in MAXQDA (VERBI GmbH Berlin, 2020) to code and re-code themes until saturation was achieved [23]. The Institutional Review Board exempted this research from review because we collected data in such a way that participants’ identities cannot be ascertained directly or indirectly via personal identifiers.

## 3. Results

### 3.1. Influencers Had Diverse Backgrounds within and Beyond the Scientific Health Community

**Figure 1:**
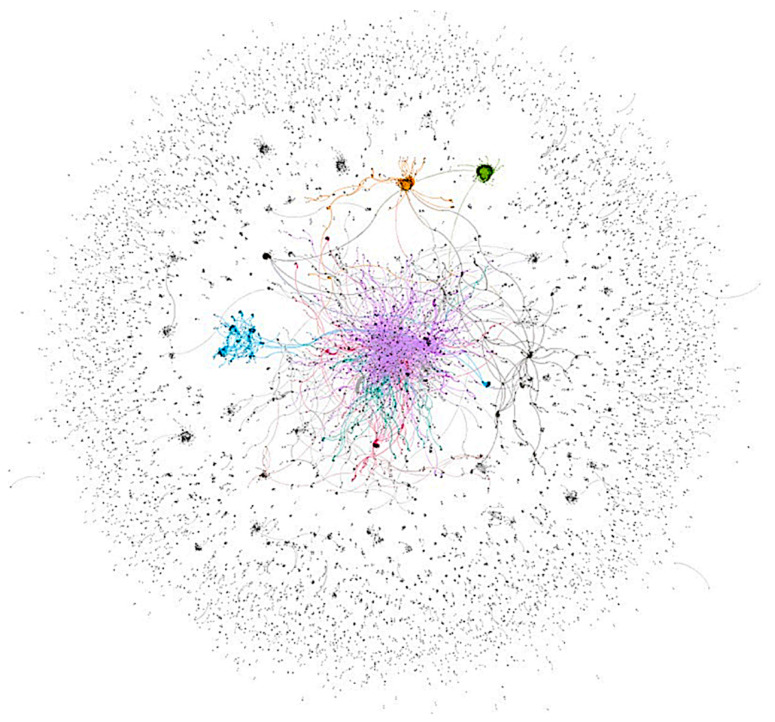
Sociogram of the overall network structure. Legend: *n* = 10,694. Each dot (node) represents a unique user, and the lines (edges) between the nodes reflect exchanged tweets (mentions and retweets). Color clusters indicate sets of users, called communities, who were algorithmically determined based on how frequently users interact together [24]. Within one community, individuals communicate with each other more frequently than with individuals outside their community.

Our analysis identified an overall social network totaling 10,694 unique users who tweeted, commented, or were mentioned at least once in breastfeeding-related tweets during the six-month period. Overall activity is visualized in the total sociogram (Figure 1) which shows: (a) distinct groups of users (called communities and differentiated by color) who interacted together frequently, (b) an interconnected network of users who spanned various communities, and (c) less engaged peripheral users around the map’s core. Breastfeeding conversations were disproportionately dominated by 813 influencers whose number of followers, number of users they follow, and number of years on Twitter varied widely (Table S4). Influencers had a wide range of professional backgrounds that were not limited to the academic, clinical, or public health spheres (Table 1). For example, while around 40% of influencers were from the scientific health community, more than half (54.7%) were from the general community such as individuals from the lay public. Commercial influencers comprised no more than 3%, out of which less than 1% were companies selling breastfeeding-related merchandise. 

### 3.2. Influencers Use Twitter for Dissemination, Advocacy, and Community Building

**Figure 2:**
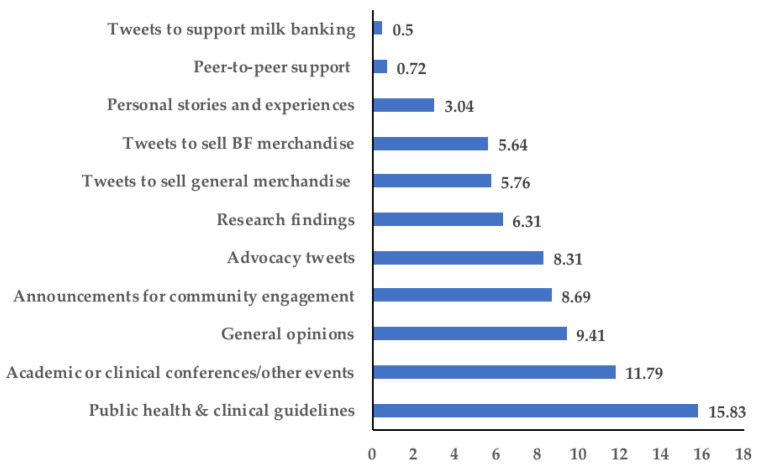
Distribution of influencers’ tweet content (*n* = 1373 tweets).

Influencers tweeted or were mentioned in tweets with widely varied content of an academic, clinical, advocacy, personal, or commercial nature (Figure 2). While most of the influencers were members of the general community, it was the scientific health community that contributed to the largest number of tweets (*n* = 798 tweets; Table S5 and Figure S2). Additionally, the type of content shared was significantly different across the three influencer groups (*p* < 0.001, chi-squared test). Specifically, the scientific health community more frequently disseminated public health/clinical guidelines, research findings, and educational updates (e.g., conference announcements). However, influencers from the general community were mostly active tweeting their opinions about the current states of both breastfeeding research and advocacy (Table S2 for examples), followed by tweets to advocate for breastfeeding-supportive environments at large. Finally, company influencers tweeted much less frequently (*n* = 20 tweets) to sell merchandise or disseminate clinical guidelines. Collectively, these findings suggest Twitter may not only be used by influencers as an information dissemination tool but also as an interactive platform for advocacy and community engagement. To examine influencers’ communication strategies, motivations, and challenges on Twitter, we analyzed 20 interviews with a purposeful sample of influencers (Table S6) who were influential for at least one month out of the six-month study period.

### 3.3. Influencers Seek Career Opportunities, Community Support, and Impact on Breastfeeding Practices via Twitter

**Figure 3:**
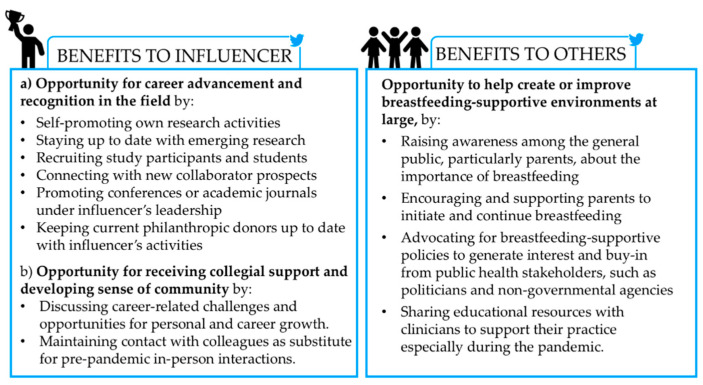
Summary of influencers’ motivations and goals when tweeting about breastfeeding. Legend: Images titled Winner (**left**) and People (**right**) by Wardehpillai and Tuk Tuk Design, respectively, from the Noun Project. Note that whether these benefits are conferred is beyond the scope of this manuscript.

Influencers’ motivations to tweet about breastfeeding can be categorized into three main themes based on *perceived* benefits to the influencer him/herself or benefits to others (Figure 3). First, many influencers, especially researchers, viewed Twitter interactions as a way to build professional connections for career advancement opportunities, as reflected in this commonly reported idea,

“I see it [Twitter] as like not only a place to share information, but also for people to know who I am. It’s helpful to have people know your name, whether it’s domestic for grants or internationally for speaking engagements.”

(Academic clinical researcher and nurse, North America)

Second, most influencers viewed Twitter as a professional platform to build collegial support and a sense of belonging and community among like-minded people, especially during the pandemic when in-person networking was not possible. Reflective of this notion, one lactation consultant from Europe said,

“I think when you work in breastfeeding, it can be a very isolated profession, particularly if you work in an area where you have a very strong bottle feeding culture…And because you’re always working where things are atypical for your population, you know, you need to reach out and find your tribe, that’s very much the people that think the same as you, people that don’t think you’re mad because you happen to believe that this [breastfeeding] is really important…When you find people that think the same as you, it can be very supportive…it can feel very collaborative.”

Third, all interviewees discussed that tweets were aimed at helping create or improve breastfeeding-supportive environments in various ways: Some took a neutral approach (due to personal preference or organizational policies) stating that they contributed by disseminating the scientific literature in hopes of helping parents make better informed infant feeding decisions. Others took an advocacy approach, such as actively promoting or lobbying for breastfeeding-supportive policies. Two quotes representing these different approaches follow:

“We want to provide hopefully neutral and fact-based statements that are easy to understand as [much as] possible, and then let people make their own decisions based on that…Advocacy is something that we [as an organization] really can’t participate in. That’s just not something that we’re allowed to do [due to an organizational policy]…We work really closely with them [national advocacy organizations] to promote and support breastfeeding. We support them through technical assistance and advice... But it’s really difficult for us to be able to retweet their content or to be able to highlight them in our social media content because of that advocacy piece.”

(Governmental health-related organization, North America)

and

“With Twitter, we’re able to reach some of our partners that maybe aren’t as invested in breastfeeding as we are, like [names of federal departments involved in labor and commerce]. So [our focus on Twitter] is on organization-to-organization advocacy.”

(State-wide breastfeeding coalition, North America)

### 3.4. Influencers Use Specific Brand Messaging Techniques That They Believe Resonate with Their Target Audiences

**Figure 4:**
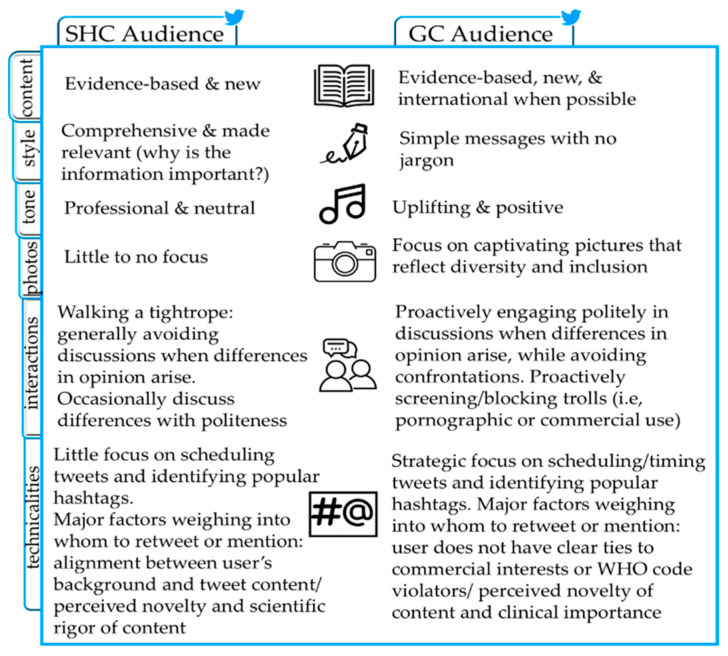
Summary of influencers’ communication strategies by type of audience. SHC, scientific health community. GC, general community. Images from top to bottom titled Book, Pen, Music, Camera, People, and Hashtag by Tippauan, Pictohaven, Iga, Yantianis, Farias, and Triyana, respectively, from the Noun Project.

During the interviews, influencers reported shaping their messaging and interactions based on whether their tweets were intended to target the scientific health community or the general community (Figure 4). Briefly, the most common characterizations of tweets for the scientific health community included being neutral in tone, evidence-informed in content, and emphasizing scientific relevance in style. Tweets for the general public, however, were mostly characterized as uplifting and positive, originating from trusted sources such as the World Health Organization, and emphasizing simplicity in wording as well as diversity in pictures. All influencers suggested discussing differences in opinion with their audience politely without any confrontations. However, influencers were more likely to intentionally take a passive approach, keeping controversial discussions to the bare minimum, when communicating with the scientific health community, as reflected in this representative quote:

“There are many controversial topics in the breastfeeding [science] world, including real and perceived conflicts of interest. It’s easy to get into heated online conversations about these issues, but I personally don’t have the energy for extensive debating on Twitter.”

(Academic researcher)

Additionally, influencers who target the general community, especially non-governmental organizations, seemed to be more strategic, such as scheduling tweets to be sent at a time when their audience is most likely to be active on Twitter or using their own created hashtags to create a campaign-like feel for advocacy and generate public interest.

Finally, independent of target audience, we noted a difference among interviewees in whether and how the WHO International Code of Marketing of Breast-milk Substitutes (WHO code) influences their communication. The WHO code aims to protect and promote breastfeeding and to ensure proper use of breast-milk substitutes when necessary, on the basis of adequate information and appropriate marketing and distribution. For example, one of the articles in the WHO code states, “Manufacturers and distributors should not distribute to pregnant women or mothers or infants and young children any gifts of articles or utensils which may promote the use of breast-milk substitutes or bottle-feeding.” In our study, one group of interviewees, mainly lactation consultants and non-governmental organizations focused on breastfeeding advocacy, voiced their strong commitment towards the WHO code. This commitment was manifested by not retweeting or mentioning any user believed to violate the code or that was financially associated with an organization believed to violate the code (perceived or actual violation). On the other hand, few influencers voiced concern about the potential negative impact of an ongoing debate within the breastfeeding research and practice field related to the perceived scope and interpretation of the WHO code.

“Sometimes I’m retweeting some scientific publications or research funded by companies [related to breastfeeding] because I think that it would be interesting for other people and even for mothers. But it’s true that in the breast milk feeding or breast milk [research and practice] area, the WHO code has huge relevance. I accept the rules [in the code] and I’m agree with them. But the point that I hate is when people use these rules to put on a fire [create a hostile environment on Twitter] and maybe to create a society that is divided into yes or no [you either agree with all aspects of the WHO code or with none]. At the end of the day, we [should] have a lot of grace. This is not a black-or-white situation. And I don’t want people who may view that rules simplistically to pressure other people [who don’t]. I mean I am part of different lactation working groups [to promote breastfeeding] but also believe sometimes research done or funded by companies provide interesting scientific evidence [innovations related to breast milk research]. I don’t want to be part of this war between companies and breastfeeding advocates. I think in equilibrium, we can find good things.”

(Academic biomedical researcher from Europe)

Taken together, influencers seem to be intentional in their message targeting approaches, which may prove applicable and valuable for other researchers and public health stakeholders who seek to establish or improve their social media presence.

### 3.5. Influencers Face Technical and Communication Challenges

We identified two main challenges that influencers generally faced when communicating about breastfeeding on Twitter. First, influencers identified limited resources (time, technology, support for hiring new staff) and technical knowledge (e.g., how Twitter algorithms help optimize one’s number of followers) as barriers to sustaining social media activity over time or for their colleagues to develop a strong Twitter presence. Reflective of this barrier, a communication specialist at a state-wide coalition explained why the coalition seeks to educate breastfeeding advocates about effective and simple social media strategies, saying,

“The other thing [barrier] in the breastfeeding sphere especially, is understanding the tech barrier that many of our most passionate advocates face. Because when you can only access your clients’ community or your peers’ community through your antiquated work technology, and because you work at a state agency that’s not well-funded, then you start to think there aren’t a lot of options [to make an impact on Twitter].”

Second, from an interpersonal communications perspective, several influencers whose target audience includes breastfeeding mothers acknowledged the challenge many users face to advocate breastfeeding without making non-breastfeeding mothers feel guilty or by coming across as judgmental. For example, one communication specialist at an international non-governmental agency that advocates for children’s rights said,

“I have noticed when I post tweets about breastfeeding, that occasionally you’ll get the women who write “ Don’t make me feel worse. And I already feel that I can’t breastfeed.” I understand where they’re coming from. It’s sad, and I respect it. But, I can’t deal with everybody’s sensitivities for the greater good.”

On the contrary, a lactation consultant and breastfeeding advocate from Europe felt more confident, saying

“We get trained from day one about listening, about communication skills. We never say to the mother, “you’re doing things wrong.” We listen, and we can always find something positive, and then we build on that connection. And so I’m very, very careful about how I phrase things. I never want to make someone feel guilty or bad by what I say. But I want to say what’s actually scientifically correct. Now some people will take offense at the scientific evidence, and I can’t change their minds. All in all, this really informs my messages and my tone and my language which can be tricky on Twitter.”

Collectively, these findings suggest that improving effective communication around breastfeeding on social media may require investment in educating public health stakeholders about best practices on social media. Specific to the field of breastfeeding research or practice, improving interpersonal skills in the virtual space may be key. 

## 4. Discussion

In this study, we identified influencers who had disproportionate influence on breastfeeding conversations on Twitter using quantitative social network analysis, and then determined what, why, and how they communicate with their audiences through a mixed-methods analytical approach. Our overarching goal was to better underscore what comprises effective public health communication strategies on social media—an important component of the 10 EPHS [1].

First, we report practical communication strategies that may contribute to being influential on Twitter. Consistent with business-to-business versus business-to-consumer marketing approaches [25], influencers used functional appeals when targeting messages for stakeholder engagement, such as tweeting about the value proposition of their research programs. On the other hand, emotional appeals were more common when influencers targeted the general public, such as encouraging mothers to breastfeeding during the pandemic using pictures of happy mothers and babies. While “breastfeeding” is not a commodity from a marketing perspective, it is interesting to observe these differences in messaging which highlight the motivations, and potential challenges in using Twitter as a public health communication platform.

From one side, Twitter has become an advocacy platform [26] in this case to promote breastfeeding-friendly policy change and to encourage and support parents to breastfeed. From another angle, influencers use the same breastfeeding Twitter hashtags for either career advancement purposes or for merely disseminating recent scientific publications without any advocacy component. While this versatility of Twitter use is in many cases an advantage to support public health communication (e.g., attracting a diverse audience, information is easily accessible and free), it seems to pose several challenges, including the strong emotions surrounding “sensitive” topics such as breastfeeding [27], that may be worth investigating in future studies.

Future research should address the following questions inspired by our findings: (a) To what extent is the difference in influencers’ motivations contributing to mixed and confusing messaging about breastfeeding science and guidelines to the public? (b) Is it possible that influencers intentionally avoid sharing important public health research findings to avoid highlighting the achievements of colleagues with competing research agendas? (c) Is it possible that influencers who are passionate breastfeeding advocates overstate research findings and use language that does not accurately reflect the underlying strength of causal inferences between mode of infant feeding and a health outcome? A practical example of this scenario would be someone tweeting that “antibodies in breastmilk prevent infants from getting COVID-19” when the current state of the evidence shows antibodies are present in breastmilk and may potentially reduce COVID-19 risk among breastfed infants.

While we did conduct detailed content analysis of tweets, one limitation is that we did not capture misleading statements which are not uncommon [28]. Similarly, we did not capture prevalent disparities between the strength of language used on social media and the underlying strength of causal inference in peer-reviewed publications [29]. Within the breastfeeding research or practice field, we suggest these aforementioned questions cannot be answered without addressing the evident differences among influencers in their commitment to, interpretation of, and reliance on the WHO code, when making strategic communication decisions on social media. Additionally, given the large role the interpretation of the WHO code plays in many of influencers’ communication decisions, it is important to note that six of the influencers we invited for an interview did not respond to the invitation. We wonder whether they may have viewed our research team as having a conflict of interest due to our research funding source. One influencer from Europe did respond back as follows “Thanks for contacting me. Unfortunately I cannot participate as I am required to comply with the WHO code. All the best with your work”. As most of our interviewees were from Europe and North America, it would be interesting to explore whether the scientific community faces similar communication challenges in other continents, and whether a study with a larger number of interviewees would identify additional challenges. 

From a methodological standpoint, this paper provides important contributions to the field of public health communications. First, we used a mixed-methods design which is only recently becoming increasingly adopted as an approach to understand supports and constraints of public health communications. For example, others have described social media activity using simple metrics such as number of likes or number of followers [30,31,32,33]. However, quantitative study designs alone overlook nuanced differences in why and how people communicate to address interpersonal or ideological challenges they may be facing within their research or practice fields. This information is important to not only understand the breadth of communication challenges, but also their depth and unique causes that may be consequential for the efficient and accurate dissemination and uptake of scientific information [34,35]. Second, by using SNA, we and others are able to make visible the invisible social influence on social media that is not reflected in more traditional social media metrics [36,37,38]. Typically, influencers are identified using arbitrary cut-offs for the number of followers. However, our social network theory-grounded findings here and elsewhere [36,38] suggest that influence goes beyond just counting to involve the networks of socially influential users. Therefore, using increasingly accessible tools, public health professionals can use real-time interactions on Twitter and potentially other social media channels to identify unique users. These users hold high demonstrable potential to impact public health messaging in today’s digital era.

## 5. Conclusions

Our findings suggest a variety of practical communication strategies and a unique and robust set of methods that public health professionals and researchers may use to support their communication strategies on social media, as means to improve one’s professional support community and public health messaging as well as to carefully study the topic. Complex challenges exist and need to be addressed for better impact on breastfeeding communication on Twitter. Beyond social media literacy and technological limitations, deep ideological differences related to influencer’s interpretation of the WHO code and which social media interactions encompass conflicts of interest may be hampering constructive communication efforts to public health stakeholders and the public. These challenges, which may also exist within other public health professions, may extend beyond social media literacy and technological limitations. Not attending to the social network data that are present on social media is to miss an enormous practice and research opportunity. Our findings have implications for other areas of exposure-related preventative public health because issues of real and perceived conflict of interest are not unique to the breastfeeding research or practice field. In fact, they are known to contribute to public skepticism, as well as slow and inaccurate public health communications [28,29].

## Figures and Tables

**Table 1:** Breakdown of influencer types.

Type of Influencer, *n* = 813	*n*	Percent
**Scientific health community**	**347**	**42.7**
clinician	125	15.4
health-related NGO	77	9.47
researcher without clinical duties	32	3.94
researcher with clinical duties	31	3.81
academic journals or conferences	17	2.09
public health employee	14	1.72
university	12	1.48
hospital or clinic	12	1.48
health-related gov. agency	12	1.48
professional association	6	0.74
others ^1^	9	1.11
**General community**	**445**	**54.7**
individual from general public	315	38.8
non-gov. non-health celebrity	85	10.5
non-health-related news	15	1.85
non-health-related NGO	11	1.35
elected government official	8	0.98
non-health-related gov. agency and programs	3	0.37
others ^2^	8	0.98
**Companies**	**21**	**2.59**
selling products unrelated to BF	15	1.85
selling products related to BF	6	0.74

NGO, non-governmental agency. Gov, governmental. BF, breastfeeding. ^1^ Such as account for a continuing medical education (CME) course. ^2^ Such as an account for a comedy show. Gray categories indicate the three broad coded influencer groups.

## Data Availability

Data available on request due to privacy restrictions. The data presented in this study are available on request from the corresponding author. The data are not publicly available due to the possibility that anyone can identify Twitter influencers by simply copy/pasting an individual tweet in Twitter’s search box, if the individual tweets are provided.

## Supplementary Materials

The following are available online at https://www.mdpi.com/article/10.3390/ijerph18126181/s1. Figure S1: The 10 Essential Public Health Services, Table S1: Codebook to describe influencer types, Table S2: Codebook to describe tweet content related to breastfeeding, Table S3: Sample interview questions, Table S4: Descriptive statistics of influencer Twitter accounts, Table S5: Differences in tweet content sent across the three influencer groups, Figure S2. Differences in influencer groups across the different tweet categories, Table S6: Basic description of interviewed influencers.
