# Supplementary material for: Breastfeeding Communication Strategies, Challenges and Opportunities in the Twitter-Verse: Perspectives of Influencers and Social Network Analysis
Source: Int J Environ Res Public Health. 2021 Jun 8;18(12):6181. doi: 10.3390/ijerph18126181 (PMC8230232; doi:10.3390/ijerph18126181)
Supplement: Supplementary file 1 [file ijerph-18-06181-s001.zip › ijerph-1237499-supplementary.pdf]

International Journal of
Environmental Research and Public Health

MDPI

Figure S1. The 10 essential public health services (retrieved from https://www.cdc.gov/publichealthgateway/publichealthservices/essentialhealthservices.html)

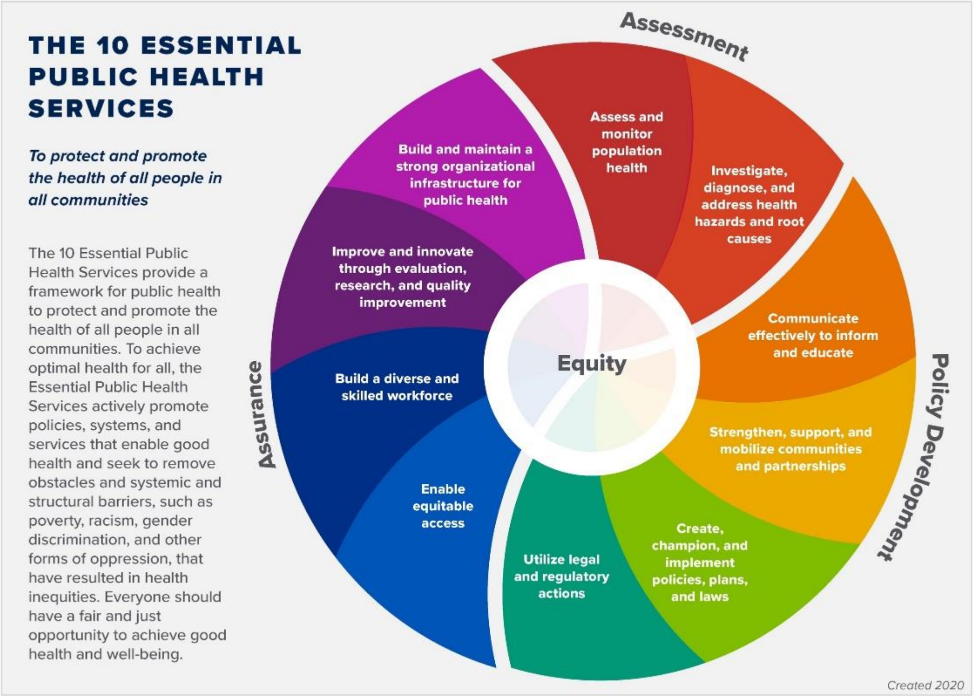

Table S1. Codebook to describe influencer types

Code	Child code	Summary title	Details
1		**Scientific health community**	
	a	researcher without clinical duties	User with a verified research-focused position, such as research professor or postdoctoral fellow **without** any clinical appointments.
	b	clinician	User with verified clinical appointment related to breastfeeding or maternal/infant health such as pediatrician, gynecologist, lactation consultant, and nurse.
	ab	researcher with clinical duties	User with a verified research-focused position such as research professor or postdoctoral fellow **with** a clinical appointment.
	c	public health employee	User who works in the public health sector, such as a public health communication officer or public health community educator
	d	university	User is managing the account of a university, university department, or institute, including medical schools
	e	hospital or clinic	User is managing the account of a hospital, hospital department, clinic, or pharmacy
	f	health-related governmental agency	User is managing the account of a health-related **governmental** agency, programs or initiatives, at the national, regional, or local levels, e.g., CDC
	g	professional association	User is managing the account of a health-related professional association, which represents the interest of professional practitioners and maintains oversight of the profession's legitimate practice, e.g., through providing training and organizing conferences.
	h	academic journals or conferences	User is managing the account of an academic journal, conference, or broadcasting station focused on health news
	i	health-related NGO	User is managing the account of a health-related **non-governmental** agency, including professional coalitions, e.g., WHO, California Breastfeeding Coalition
	j	others	All other users from the scientific health community
**2**		**General community**	
	o	individual from the general public	Layperson who is **not** from the scientific health community and who does not fit any other category below
	p	non-governmental non-health-focused celebrities	User whom we assumed may be popular such as Hollywood actors
	q	non-health related news	User is managing the account of a broadcasting station or podcast or other media outlet **not** focused on health news
	r	non-health related governmental agency & programs	User is managing the account of a non- health related **governmental** agency, programs or initiatives, at the national, regional, or local levels, e.g., UK Department of Education
	s	elected government official	User with a verified non-health focused elected position in local, regional, or nation-wide government

	t	non-health related NGO	User is managing the account of a non-health related **non-governmental agency,** e.g., Glasgow Life Organization
	u	other	All other users from the general community
**3**		**Companies**	
	x	selling merchandise unrelated to BF	User is managing the account of a commercial company that sells merchandise not directly related to breastfeeding, such as baby clothes, jewelry, illustrations, and art posters of mothers and babies
	z	selling merchandise related to BF	User is managing the account of a commercial company that sells breastfeeding-related merchandise such as breast pumps, bras, books, and recipes as galactagogues

*Code and child code numbers/letters are chosen randomly to help organize the data and do not have any mathematical meaning. NGO, non-governmental organization. BF, breastfeeding.

International Journal of Environmental Research and Public Health

MDPI

Table S2. Codebook to describe tweet content related to breastfeeding

Code*	Summary title	Description	Example of tweet^
11	Research findings	Peer-reviewed research findings shared in the form of statements, links to research papers blogs, or pictures of Powerpoint slides from conferences	Published Today in @journal: The longer women #breastfeed, the more they are protected against #breastcancer.
12	Public health & clinical guidelines	Public health or clinical guidelines, recommendations, or policy briefs endorsed by academic or health organizations (governmental or non-governmental)	The #WHO recommends infant be exclusively #Breastfed for the first six months of life #nutrition #humanmilk #breastfeeding @UniversityName
13	Academic or clinical conferences/other events	Invites or academic updates related to:<br>• research talks, symposia, conferences, and workshops, or<br>• clinical training events, or<br>• grant or employment opportunities	Mark your #Calendar for June 9. Join us the #nameOfConference. Focus on #lipids in #breastmilk @cityname @university. Register HERE. [link to conference page provided]
21	General opinions	Opinions about the field of breastfeeding research or the current state of breastfeeding promotion, protection, and support	Why does #NameOfNewsStation say breastfeeding is not harmful during #pandemic? So bad for #breastfeeding cause. should say it's essential.
22	Announcements for community engagement	Invites for parents to:<br>• join support groups or awareness sessions<br>• call hotline<br>• schedule appointment at clinic<br>• participate in studies recruitments or awareness campaigns as well<br>Thank you notes exchanges between parents and stakeholders (e.g., lactation consultant, pediatrician, research, health organization)	Do you know that the @HotlineName offers #breastfeedingsupport in #TownName? Call 00000000000 [link to website provided]
23	Advocacy tweets	Tweets that celebrate or advocate breastfeeding protection, promotion, and support, such as advocating for public breastfeeding, maternity leave-supportive laws/policies/resolutions, establishing pumping rooms, or standing against breastfeeding sexualization.	Reach out to your #Congress members. Push them to support this #nameOfBill that helps #breastfeeding families with better #paid #maternity leave.
24	Personal stories and experiences	Personal stories and experiences related to breastfeeding	That moment when you are #breastfeeding at 2 am and all you want is a big bowl of #strawberries! [includes selfie of mother and baby]

25	Tweets to support milk banks	Tweets that encourage others to donate milk or funds to a milk bank	Every #breastmilk drop matters. We are accepting @milkbankname today and help us save #preterm babies.
26	Peer-to-peer support	Tweets that provide or ask for advice, recommendations, tips, or resources related to how to support parents in their breastfeeding journey. These tweets address fellow clinicians such as lactation consultants.	Any tips on how to conduct a successful #Zoom call with #breastfeeding mothers? Never done this before. Want mom to feel #safe and #comfortable. #IBCLC @HospitalName
31	Tweets to sell merchandise related to breastfeeding	Advertisement for purchasing breastfeeding-related merchandise such as breast pumps, bras, books, and recipes as galactagogues	So easy to #breastfeed with the new #nameOfBreastPump edition! Makes a perfect #gift for #babyshower. [link to store included]
32	Tweets to sell general merchandise	Ad for purchasing merchandise not directly related to breastfeeding such as baby clothes, jewelry, illustrations, and art posters of mothers and babies	Available now! Our new collection of #baby clothes for Spring 2020 is out. Check out the cutest outfits for your #breastfeeding baby here [link to store included]

[*]Code numbers are chosen randomly to help organize the data and do not have any mathematical meaning. [^]Tweets are not real but inspired by actual tweets to ensure influencers remain anonymous because copy/pasting a tweet into the Twitter "search box" will likely identify the tweet itself and its sender. We excluded tweets that used breastfeeding hashtags for pornographic purposes or for irrelevant purposes, such as tweeting about one's breastfeeding cat.

Table S3. Sample interview questions

1) What are the reasons you tweet around breastfeeding? What goals do you want to achieve by engaging in Twitter discussions related to breastfeeding?
2) What topics do you tweet about?
3) How do you decide what to tweet, retweet and what to comment on?
4) What drives your decision not to tweet, retweet, or comment about certain topics related to breastfeeding?
5) How do you decide whom to follow on Twitter?
6) Do you know who your audience is? Who is it comprised of? Who is your target audience?
7) What do you think resonates most with your followers in terms of tweet content, language and tone?
8) What strategies do you use on Twitter that you believe are helping you reach your target audience?
9) What challenges do you personally face when communicating on Twitter?
10) From a technical standpoint, is there anything you would like to learn (e.g., how Twitter works, different tools) that might help you achieve your communcation goals?

Table S4. Descriptive statistics of influencer Twitter accounts

	Median	IQR	Range
Status count	39,757	128,106	512 to 421,393
Followers count	138,698	1,063,525	234 to 19,398,870
Favorites count	3002	12,006	0 to 43,178
Friends count	814	1605	46 to 13,513
Age of account, years	11	3	2 to 14

*More than 75% of influencers were from North America or Europe. IQR, interquartile range. *n*=813.

Table S5. Differences in tweet content sent across the three influencer groups

Tweet category*	Scientific community (n=798 tweets)	General community (n=233 tweets)	Companies (n=20)
Research findings	70 (8.77)	28 (12.0)	2 (10)
Public health & clinical guidelines	168 (23.1)	29 (12.4)	6 (30)
Academic & clinical conferences	93 (12.8)	10 (4.29)	0
General opinions	43 (5.91)	71 (30.5)	0
Community engagement	126 (17.3)	20 (8.58)	1 (5)
Advocacy tweets	76 (10.4)	39 (16.7)	2 (10)
Personal stories & experiences	12 (1.65)	27 (11.6)	1 (5)
Tweets to support milk banking	6 (0.82)	3 (1.29)	0
Peer-to-Peer support	11 (1.51)	1 (0.43)	0
Tweets to sell BF merchandise	91 (12.5)*	5 (2.15)	6 (30)
Tweets to sell general merchandise	102 (14.0)	0	2 (10)

* Tweet category differences across the three influencer groups are statistically significant (*P*<.001; Chi-squared test).

Values are n (%) where % the percentage of tweets within one influencer category. For example, 70 tweets were sent by the scientific community influencers related to research findings, which represents 8.77% of all tweets sent by influencers from the scientific community. * The 12.5% of tweets to sell general merchandise by the scientific health community may be misleading, as these tweets were all sent by one user only.

Figure S2. Differences in influencer groups across the different tweet categories

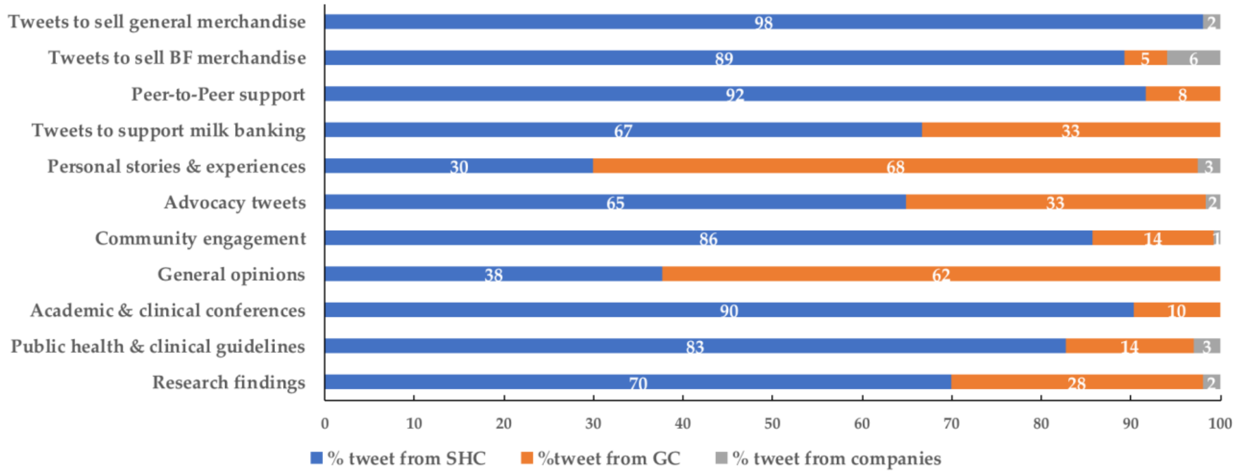

SHC, scientific health community influencers. GC, general community influencers. Unlike Table S5 that shows percentage of each tweet category by influencer type, Figure S2 shows the distribution of influencers by tweet type. For example, 70% of tweets about research findings were sent by the SC compared to 28% by the general community.

Table S6. Basic description of interviewed influencers

Number of interviewees	Description of Influencer*	Geographical Area
1	Academic epidemiology researcher	North America
1	Academic biomedical researcher	Europe
1	Academic clinical researcher and nurse	North America
1	Academic translational researcher and pharmacist	Australasia[1]
1	Academic clinical researcher and gynecologist	Europe
1	Lactation consultant and journal publisher	Australasia
1	Academic clinical researcher, lactation consultant, and book editor	North America
3	Lactation consultant	Europe
1	Lactation consultant and director of state-wide breastfeeding coalition	North America
2	Public health professional with communication or directorship roles at an *international non-governmental agency*	Asia or North America
2	State-wide breastfeeding coalition	North America
1	Health-related *federal governmental* agency	North America
1	Health-related *nationwide non-governmental* agency^	North America
1	Health-related *nationwide non-governmental* agency^	Europe

1	Health-related *international non-governmental* agency	International; account managed from North America
1	Journalist who covers African Women's issues	Africa

[*] 2 (20%) of interviewed influencers were male. [^] account managed by volunteer. [1] Australasia covers Australia, New Zealand and a few neighboring islands.
